# Frontal QRS–T angle remains unchanged in fibromyalgia: a cross-sectional study with implications for routine ECG screening

**DOI:** 10.3389/fmed.2025.1615561

**Published:** 2025-07-08

**Authors:** Hüseyin Tezcan, Ezgi Akyıldız Tezcan

**Affiliations:** ^1^Department of Cardiology, Faculty of Medicine, Selcuk University, Konya, Türkiye; ^2^Department of Physical Medicine and Rehabilitation, Faculty of Medicine, Selcuk University, Konya, Türkiye

**Keywords:** fibromyalgia, frontal QRS-T angle, electrocardiogram, arrhythmia, chronic pain, autonomic dysfunction, duloxetine, cardiac screening

## Abstract

**Aim:**

Fibromyalgia syndrome (FMS) is a chronic condition characterized by widespread pain and associated with systemic diseases. Although autonomic dysfunction in FMS may predispose to cardiac arrhythmias, its impact on cardiac parameters, such as the frontal QRS-T [f(QRS-T)] angle, remains unclear. This study aimed to investigate the f(QRS-T) angle in FMS patients compared to healthy controls.

**Methods:**

A total of 75 FMS patients and 75 healthy controls were included in this cross-sectional study. Disease severity was assessed using the Fibromyalgia Impact Questionnaire (FIQ). The f(QRS-T) angle was calculated from 12-lead electrocardiograms by a blinded cardiologist. Associations between the f(QRS-T) angle, FIQ scores, and duloxetine use were analyzed using appropriate statistical methods.

**Results:**

No significant differences in the f(QRS-T) angle were observed between FMS patients and controls (*p* = 0.973). Additionally, no correlation was found between FIQ scores and the f(QRS-T) angle (*p* = 0.725). Subgroup analysis revealed no significant differences in the f(QRS-T) angle between FMS patients using duloxetine and those not using it (*p* = 0.503).

**Conclusion:**

Contrary to concerns about subclinical cardiac involvement in FMS, our findings reveal no significant alterations in the f(QRS-T) angle among FMS patients. Moreover, disease severity and duloxetine use do not influence this parameter. These results challenge the assumption of clinically relevant cardiac dysregulation in FMS and suggest that routine ECG screening may not be necessary for patients with FMS. Nonetheless, longitudinal studies are warranted to fully clarify the long-term cardiac risk in this population.

## 1 Introduction

Fibromyalgia syndrome (FMS) is a prevalent medical condition characterized by persistent, widespread pain that affects multiple areas of the body and endures for more than 3 months (1, 2). In addition to chronic pain, patients commonly experience a range of other symptoms—including fatigue, sleep disturbances, and cognitive or somatic issues—that profoundly impact quality of life and often lead to significant physical and psychological distress (2).

Beyond these hallmark symptoms, FMS is known to be associated with a variety of clinical conditions such as chronic fatigue syndrome, irritable bowel syndrome, interstitial cystitis, and inflammatory and degenerative rheumatic conditions (3). Although FMS is recognized to involve multisystem dysregulation, the precise relationship between FMS and cardiac pathologies remains an underexplored area of research. This gap in knowledge clearly underscores the need for in-depth investigations into the cardiac implications for patients with FMS.

In the realm of cardiac diagnostics, the electrocardiogram (ECG) stands as a critical and versatile tool. Its cost-effectiveness, non-invasiveness, rapid execution, and immediate result availability make it invaluable in stratifying patients for various cardiac conditions and assessing overall mortality risks (4). Within ECG analysis, the frontal QRS-T [f(QRS-T)] angle, which quantifies the angular disparity between the QRS complex and T wave axes, has emerged as a significant metric. Its association with increased risks of arrhythmias, all-cause mortality, and sudden cardiac death underlines its clinical importance (4, 5). The f(QRS-T) angle’s straightforward calculation from standard surface ECGs renders it an especially practical parameter in clinical settings.

In the context of FMS, existing studies have identified several ECG abnormalities, including increased P wave dispersion, and variations in QT interval duration (6–9). These findings imply potential cardiac dysfunctions in FMS patients, potentially linked to underlying autonomic dysfunction, and suggest an elevated risk of cardiac arrhythmias. Emerging evidence shows that autonomic tone, although not the primary determinant, measurably modulates the frontal QRS-T angle. In type 2 diabetes, patients with confirmed cardiovascular autonomic neuropathy display a significantly wider spatial angle that correlates inversely with heart-rate-variability indices (10), and an enlarged angle predicts long-term mortality as powerfully as formal autonomic testing (11). High-resolution Holter studies further demonstrate that sympathetic surges widen, whereas vagal predominance narrows, the angle on a beat-to-beat basis (12). Taken together, these data indicate that the QRS-T angle chiefly reflects myocardial electrical heterogeneity but is secondarily shifted by autonomic imbalance, justifying its use here as an indirect marker of the dysautonomia thought to underlie arrhythmic risk in FMS. However, the f(QRS-T) angle specifically has yet to be systematically examined in a larger, more representative FMS cohort. This represents a significant lacuna in current research.

Our study aims to bridge this gap described above by comparing the f(QRS-T) angle of FMS patients and healthy controls. Through this comparison, we aim to enhance the understanding of cardiac health in FMS and potentially contribute to improved diagnostic and therapeutic strategies for individuals affected by this condition. Given the rising prevalence of FMS and its systemic impact, our study’s insights into cardiac parameters hold significant clinical relevance.

## 2 Materials and methods

### 2.1 Study design and participants

This cross-sectional, prospective study received approval from the local ethics committee of the medical faculty (Approval number: 2023/4489), adhering to the principles of the World Medical Association Declaration of Helsinki (2000, Edinburgh). Written informed consent was obtained from all participants.

From September 2023 to December 2023, consecutive FMS patients in follow-up at the physical medicine and rehabilitation outpatient clinic and healthy volunteers were enrolled. Inclusion criteria encompassed participants aged from 18 to 65, and for FMS patients meeting American College of Rheumatology diagnostic guidelines (13). Exclusion criteria comprised individuals with a history of specific conditions, such as coronary artery disease, dysrhythmia, heart valve disease, hypertension, heart failure, pregnancy, malignancy, major kidney or liver disease, uncontrolled diabetes mellitus, hypothyroidism, and other severe endocrine disorders. Participants were meticulously selected based on stringent inclusion and exclusion criteria, ensuring a representative sample of the FMS population.

The control group, consisted of individuals attending outpatient clinics unrelated to FMS. Control group participants were briefed on the study’s scientific relevance and screened to ensure the absence of FMS-related symptoms or examination findings. Demographic and clinical features were recorded.

### 2.2 Assessment tools

The impact of FMS was assessed using the Fibromyalgia Impact Questionnaire (FIQ), a validated 10-item self-administered questionnaire adapted into Turkish used by Sarmer et al. (14, 15). The FIQ comprises various dimensions, including physical function, general wellbeing, work capacity, and symptoms of pain, fatigue, stiffness, anxiety, and depression. The resulting total score ranges from 0 to 100, with higher scores indicating more severe disease.

### 2.3 Electrocardiogram examination

**FIGURE 1:**
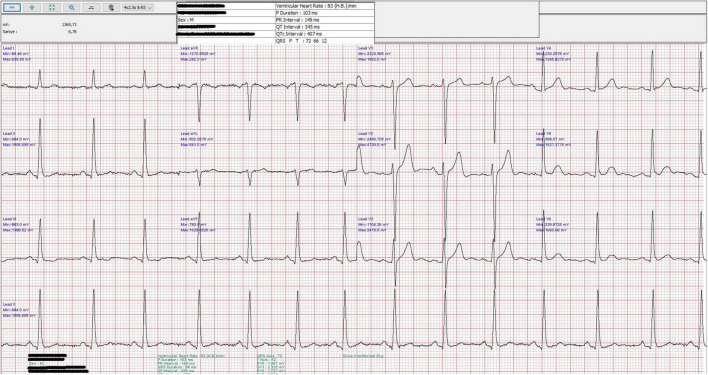
A standard 12-lead electrocardiogram (ECG) showing sinus rhythm with a heart rate of 83 bpm. The QRS axis is 72° and the T axis is 12°, resulting in a frontal QRS-T angle of 60°, calculated as the absolute difference between the QRS and T axes (|QRS axis–T axis|).

In this study, each participant underwent a 12-lead ECG test while resting, conducted using the Biocare IE12A device, provided by Shenzhen Biocare Biomedical Equipment in China. Patients were positioned supine for the duration of the ECG recording. The procedure was standardized with the ECG machine set to a paper speed of 25 mm/s, a voltage amplitude setting of 10 mm/mV, and a broad filter range spanning 0.16–100 Hz. All ECG recordings were standardized in terms of patient positioning, time of day, and technician training, to minimize variability and enhance the reliability of our findings. A cardiologist, who was blinded to patient details, conducted a thorough analysis of each participant’s 12-lead ECG. The focus of the analysis was on the frontal QRS-T angle, defined as the absolute difference between the axis of the QRS complex and T wave in the frontal plane (Figure 1) (4). For cases where this angular difference exceeded 180 degrees, it was recalculated by deducting the value from 360 degrees.

### 2.4 Sample size determination, over-recruitment strategy and final sample

*A priori* power analysis (G*Power 3.1.9.7) indicated that 64 participants per group would detect a medium effect (d = 0.50, α = 0.05, 1–β = 0.80). To offset unforeseen exclusions we planned to enroll 90 participants per group and analyze the first 75 eligible subjects in each arm.

### 2.5 Statistical analyses

Statistical analyses were performed using SPSS version 22.0 (IBM, Armonk, New York, United States). Categorical variables were presented as numbers and frequencies, while mean [standard deviation (SD)] and median [interquartile range (IQR)] described continuous variables. The Chi-Square test compared categorical variables. Normal distribution was assessed visually (histograms) and analytically (Kolmogorov-Smirnov). Parametric tests (independent-samples t) were applied to normally distributed variables; non-parametric tests (Mann-Whitney U) were used for variables violating normality assumptions. A significance level of *p* < 0.05 was applied, with a 95% confidence interval.

## 3 Results

**FIGURE 2:**
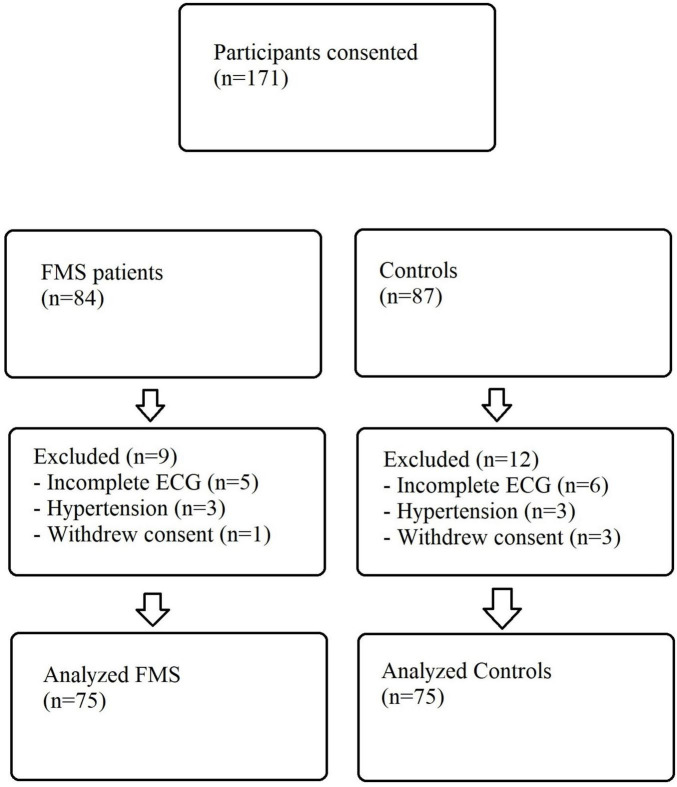
Participant-flow diagram.

Of 171 individuals who provided written consent (84 with FMS and 87 potential controls), 21 were excluded after enrolment—11 because of incomplete or unreadable ECG recordings, six owing to newly diagnosed hypertension, and four who withdrew consent—leaving 150 participants (75 FMS, 75 controls) for the final analysis (Figure 2). The preponderance of participants within our study cohort consisted of females (95%), consistent with the higher prevalence of FMS among women. No significant disparities in demographic characteristics or laboratory parameters were observed between the two cohorts. Detailed information on these variables is presented in Table 1.

**TABLE 1:** Demographic and clinical findings of participants.

Variable	FMS (*n* = 75)	Control group (*n* = 75)	*P*-value
Age (year); median (IQR)	44 (34–50)	43 (30–51)	0.668
BMI (kg/m^2^); mean ± SD	28.61 ± 5.45	28.27 ± 5.6	0.718
Gender, male; *n* (%)	3 (0.4%)	5 (0.7%)	0.719
Smoking status; smoker *n* (%)	16 (21.3%)	10 (13.3%)	0.281
Hemoglobin (g/dL); mean ± SD	13.06 ± 1.37	13.16 ± 1.44	0.647
WBC (10^9^/L); median (IQR)	7.32 (5.84–8.46)	7.61 (6.29–8.92)	0.328
Platelet (10^9^/L); median (IQR)	279 (239–333)	279 (243–320)	0.979
CRP (mg/dL); median (IQR)	3.25 (2.08–5.2)	3.65 (2.35–7.1)	0.196
ESR (mm/h); median (IQR)	10 (6–13)	10 (5–15)	0.980
AST (U/L); median (IQR)	18 (15–22)	18 (15–22)	0.868
ALT (U/L); median (IQR)	16 (12–21)	15 (13–22)	0.895
BUN (mg/dL); median (IQR)	26 (22–31)	27 (21–31)	0.657
Creatinine (mg/dL); median (IQR)	0.71 (0.64–0.77)	0.68 (0.63–0.77)	0.389

ALT, alanine transaminase; AST, aspartate transaminase; BMI, body mass index; BUN, blood urea nitrogen; CRP, C-reactive protein; FMS, fibromyalgia syndrome; IQR, interquartile range; N, number; SD, standard deviation; WBC, white blood cells.

In the evaluated cohort, heart rate comparisons revealed no statistically significant differences between patients and control subjects (mean ± SD; 72.48 ± 7.87, 70.82 ± 7.53, *p* = 0.193). The QRS complex duration, showed no significant difference between FMS patients, with an average of 89.21 milliseconds (ms) and a SD of 9.57 ms, and the control group, with an average of 87.22 ± 8.66 ms (*p* = 0.186). Additionally, electrocardiogram assessments identified no cases of either right or left bundle branch block among the participants. No statistically significant distinction in the f(QRS-T) angle was observed between individuals diagnosed with FMS and those constituting the control group [median (IQR); 19 (9–31), 19 (8–34); *p* = 0.973].

**FIGURE 3:**
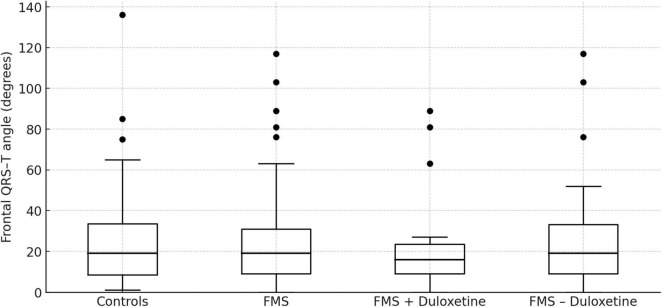
Frontal QRS–T angle in healthy controls (*n* = 75), fibromyalgia patients overall (FMS, *n* = 75), and fibromyalgia syndrome (FMS) sub-groups using (*n* = 19) or not using (*n* = 56) duloxetine. Boxes represent the inter-quartile range (IQR); horizontal lines are medians; whiskers reach the most extreme values within 1.5 × IQR; filled circles denote statistical outliers. Mann–Whitney U tests: FMS vs. controls, *p* = 0.973; FMS+ vs. –duloxetine, *p* = 0.503.

In the assessment of disease severity among FMS patients using the FIQ, the median FIQ score was determined to be 70.9 (IQR: 60.76–81.37). Notably, no significant association was identified between the FIQ score and the f(QRS-T) angle [correlation coefficient (CC) = 0.446, *p* = 0.725]. A subset of the FMS patient cohort, specifically 25.3%, reported the use of duloxetine, while others employed non-steroidal anti-inflammatory drugs on an irregular basis for pain management. Importantly, the analysis revealed no discernible distinction in f(QRS-T) angle measurements between patients utilizing duloxetine and those abstaining from it [median (IQR); 16 (7–24), 19 (9–33.75); *p* = 0.503]. f(QRS-T) angle values can be seen in Figure 3.

## 4 Discussion

This study proved that the frontal QRS-T angles in FMS patients do not significantly differ from those in healthy controls. Furthermore, our analysis reveals no correlation between these angles and FIQ scores or the use of duloxetine, suggesting that the severity of FMS symptoms and certain treatments do not notably influence this specific cardiac parameter. This finding challenges some prevailing assumptions about cardiac involvement in FMS, adding a nuanced perspective to our understanding of the syndrome’s cardiac manifestations.

For over two decades, research has intensely focused on autonomic dysfunction in FMS individuals, given its potential links to various clinical conditions (16, 17). From a cardiogenic perspective, this dysfunction is intricately linked to various clinical conditions, notably an increased susceptibility to cardiac arrhythmias and sudden death. As a result, a substantial body of research has been dedicated to examining the link between FMS and cardiac arrhythmias. Within this framework, ECG serves as a pivotal diagnostic tool for identifying predictors of cardiac arrhythmias and associated risks. Specifically, the P wave signifies atrial depolarization, the QRS axis denotes ventricular depolarization, and the T axis represents ventricular repolarization. Consequently, P wave dispersion assumes significance as a key indicator of atrial fibrillation, while QT dispersion, fragmented QRS morphology, Tpeak-Tend time, Tpeak-Tend/QT ratio, and the f(QRS-T) angle collectively serve as indicators of ventricular arrhythmias (4, 18–22).

If we look at the studies on ECG parameters in fibromyalgia patients, QT and P wave dispersions have been examined in many studies. Regarding QT dispersion, a parameter extensively evaluated in FMS patients, studies have reported inconsistent outcomes, reflecting the complexity of the condition (6–8). Yolbaş et al.’s (7) study, including 48 FMS patients and 32 healthy controls, reported diminished QT dispersion in FMS patients compared to healthy controls. Conversely, Aksu et al.’s (8) study, involving 43 FMS patients and 30 healthy controls, identified heightened QT dispersion in FMS patients relative to their healthy counterparts. P wave dispersion, another variable under scrutiny, has exhibited variability in study outcomes (7, 9). Yolbaş et al.’s (7) study observed no discernible difference, while Akkaya et al.’s (9) study, involving 70 FMS patients and 70 control subjects, corroborated an increase in P wave dispersion in FMS patients. The varying results regarding P wave and QT dispersion in FMS patients across different studies may reflect methodological variances or the complex nature of FMS. While our study did not focus on P wave and QT dispersion, the variability in these parameters in other research underscores the complexity of cardiac effects in FMS. Unlike the aforementioned investigations, Günlü et al.’s (6) study focused on FMS patients who presented with palpitations. Interestingly, despite evaluating a symptomatic subgroup, they concluded that arrhythmic risk was not heightened in FMS, and they found no significant difference in the f(QRS)-T angle between FMS patients and controls. Nonetheless, when comparing findings across studies, it is important to remember that differences in patient symptomatology—such as the presence of palpitations—may influence both clinical presentation and study outcomes.

The relationship between fibromyalgia severity and ECG parameters remains largely underexplored. Although the f(QRS)-T angle itself was not examined in previous research, the only study addressing disease severity in relation to ECG parameters was conducted by Akkaya et al. (9), who observed a correlation between FIQ scores and P wave dispersion. In contrast, our findings revealed no significant relationship between FIQ scores and the f(QRS)-T angle, suggesting that the overall impact of fibromyalgia, as measured by the FIQ, may not extend to this particular ECG parameter. Moreover, our evaluation of duloxetine—a medication with notable noradrenergic activity—showed no discernible effect on the f(QRS)-T angle. This result counters the hypothesis that noradrenergic modulation might alter the cardiac electrophysiology of FMS patients, emphasizing the need for further investigation into the mechanisms behind these observations.

Nevertheless, it is important to acknowledge the limitations inherent in our study. Its single-center design and the limited scope of the patient cohort might restrict the generalizability of our findings. It is also worth noting that 95% of our sample were female, reflecting the well-documented higher prevalence of FMS in women. While this aligns with real-world clinical distributions, it may limit the applicability of our results to male FMS patients. We did not include direct autonomic tests such as heart-rate variability, heart-rate recovery, or the COMPASS-31 questionnaire; therefore, the frontal QRS-T angle served only as an indirect surrogate of autonomic balance. Controls were recruited from outpatient clinics. Although we excluded overt cardiometabolic disorders, this convenience sampling may have introduced unmeasured factors that blunt inter-group differences. Furthermore, the cross-sectional nature of this study prevents us from drawing conclusions about the longitudinal cardiac impact of FMS. Future research should aim for a multicenter approach with a more diverse patient cohort, ideally including longitudinal analyses to assess potential temporal changes in cardiac parameters in FMS.

In this cross-sectional study of predominantly female FMS patients, we found no significant difference in the frontal QRS-T angle compared to healthy controls, nor any meaningful association between fibromyalgia severity, duloxetine use, and this ECG parameter. These findings challenge the notion of subclinical cardiac dysregulation in FMS and suggest that routine cardiac screening focused on this parameter may not be necessary in asymptomatic patients. Nevertheless, further longitudinal and multicenter investigations are essential to fully clarify the long-term cardiac risks in FMS and determine whether additional monitoring or interventions are warranted for particular subgroups.

## Data Availability

The raw data supporting the conclusions of this article will be made available by the authors, without undue reservation.
